# Supplementary material for: SARS-CoV-2 Spike Protein-Expressing Enterococcus for Oral Vaccination: Immunogenicity and Protection
Source: Vaccines (Basel). 2023 Nov 14;11(11):1714. doi: 10.3390/vaccines11111714 (PMC10675790; doi:10.3390/vaccines11111714)
Supplement: Supplementary file 1 [file vaccines-11-01714-s001.zip › vaccines-2663556-supplementary.pdf]

Table S1. Assessment of the Efficacy of Probiotic Vaccine Vac 2 in Reducing SARS-CoV-2 Virus Accumulation in Syrian Golden Hamsters' Lungs

Experimental groups	Researched Parameters	Time After Infection	
		3	6
Vac 1	Infectious Virus Titer (lg PFU/ml, mean ± SEM)	5,53±0,03	4,44±0,03
	Reduction in Virus Infectious Titer (Δ, lg)	0,02	0,75
	Inhibition Coefficient (%)	3,55	82,07
Vac 2	Infectious Virus Titer (lg PFU/ml, mean ± SEM)	5,46±0,03	2,82±0,07
	Reduction in Virus Infectious Titer (Δ, lg)	0,09	2,36
	Inhibition Coefficient (%)	19,29	99,56
E. faecium L3	Infectious Virus Titer (lg PFU/ml, mean ± SEM)	5,55±0,03	5,18±0,05

Table S2. Scoring system for pathological changes in lung

Degree of damage (points)	Pathological-anatomical changes
0 (without changes)	The lungs exhibit a normal anatomical and physiological state characterized by pale pink coloration and a lack of pronounced vascular markings. Lung volume and consistency are within the normal range, with smooth organ margins.
1 (light)	The lungs are moderately full-blooded, the vessels in the bronchial part are dilated. The edges are usually smooth, graypink in color. There are normal areas without pathologies (pink lung color) alternating with pathological focal inflammatory changes (red-gray lung color). Small (about 1 mm) hemorrhagic foci may be present. Lungs volume and consistency in most cases comply with normal value.
2 (moderate)	The edges of the lungs have a red-gray tint. Medium-sized (2-3 mm) hemorrhagic foci in both lungs are detected at the autopsy. The lungs are swollen. The consistency of the organ is somewhat flabby, in some cases testy. Focal pneumonia.
3 (moderate-severe)	Large-focal, lobar (lobar), drain (in some cases multiple) semi-painful pneumonia. The color of the pathological areas of the lungs is saturated red, or cherry red with a dirty gray tint. Hemorrhages of medium (2-3 mm) and large (more than 3 mm) size are commonly detected in both lungs. The vascular pattern is pathologically altered. The consistency is testy with a light seal.

4<br>(severe)	Diffuse, extensive pneumonia, usually involving all segments of the right and left lung. Large, draining hemorrhages in both lungs. The color of the lungs is dark red, brown-red, usually with a clear brown tinge. The consistency of the organ is compacted. The vascular pattern is not distinguishable.

Table S3. The pulmonary changes upon postmortem examination of Syrian hamsters, infected with SARS-CoV-2 virus

Groups	Changes in the lungs (m±SEM) during infection		
	Day 0	Day 3	Day 6
Vac1	0,0±0,0	0,7±0,7	1,0±0,0
Vac2	0,0±0,0	0,7±0,7	0,3±0,3
Untreated control group	0,0±0,0	0,7±0,3	1,3±0,3

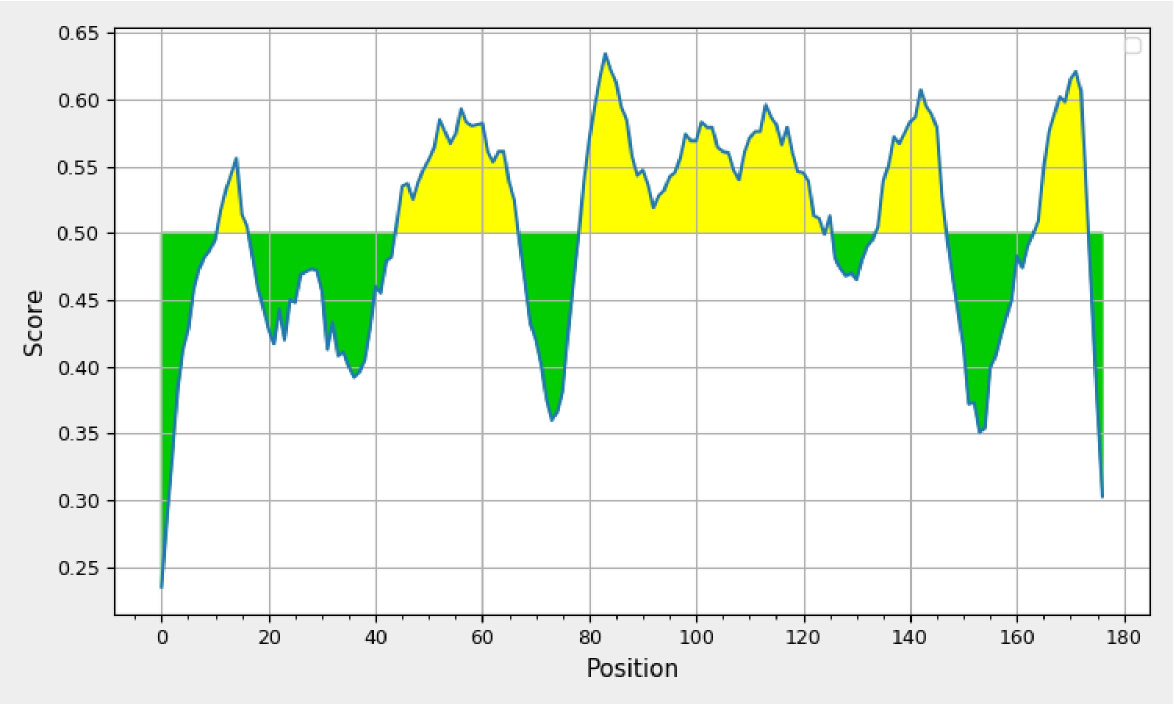

Figure S1. B Cell Epitope Prediction

BepiPred Linear Epitope prediction resources was used to predict linear B epitopes from the protein structure.

BepiPred predicted the presence of B epitopes in the amino acid sequence S1 with a threshold ranging from 0.25 to 0.634.

BepiPred threshold graph of SB protein predicting linear epitopes. Yellow color depicts the predicted epitope residues

Allel e	#	Star t	En d	Lengt h	Peptide	Core	Icore	Score	Percentil e Rank
HLA-B*07:02	1	147	154	8	QPYRVVVL	QPY-RVVVL	QPYRVVVL	0.75154	0.1
HLA-B*07:02	1	167	174	8	GPKKSTNL	GPKKSTN-L	GPKKSTNL	0.69206 3	0.13
HLA-B*07:02	1	167	176	10	GPKKSTNLEF	GPKKSTLEF	GPKKSTNLEF	0.67402	0.13
HLA-A*02:01	1	65	74	10	KLPDDFTGC V	KLPDDFTG V	KLPDDFTGC V	0.56284 1	0.23
HLA-A*02:01	1	27	36	10	KLNDLCFTN V	KLNDLFTN V	KLNDLCFTN V	0.53341 6	0.24
HLA-A*02:06	1	65	74	10	KLPDDFTGC V	KLPDDFTG V	KLPDDFTGC V	0.53299 9	0.21

Figure S2. MHC-I Binding Prediction Results. Binding Scores.

Prediction method: NetMHCpan EL 4.1 | High Score = good binder

Peptide Information:

Peptide
ALHMDYSVLYNSASFSTFKCYGVSPTKLNDLCFTNVYADSFVIRGDEVRQIAPGQTGNIADYNYKLPDD FTGCVIAWNSNNLDSKVGGNYNYLYRLFRKSNLKPFERDISTEIYQAGSTPCNGVKGFNCYFPLQSYGF QPTYGVGYQPYRVVVLSFELLHAPATVCGPKKSTNLEFY

MHC-I Alleles:
HLA-A*02:01
HLA-B*07:02
HLA- A*02:06

HLA-A*02: This is one of the most common HLA-A alleles in Europe and is found in relatively high frequencies across the continent.
HLA-B07 is also common in European populations, with varying frequencies in different regions.

Conclusion: Based on the score values, the investigated peptide contains epitopes with a strong predicted binding affinity to HLA-A02:01, HLA-A02:06, and HLA-B07:02 MHC-I. However, it is important to emphasize that while these predictions offer valuable insights, experimental validation is essential to confirm the actual peptide-MHC-I binding and immunogenicity.

References: Birkir Reynisson, Bruno Alvarez, Sinu Paul, Bjoern Peters, Morten Nielsen. 2020. NetMHCpan-4.1 and NetMHCIIpan-4.0: improved predictions of MHC antigen presentation by concurrent motif deconvolution and integration of MS MHC eluted ligand data. Nucleic Acids Res. 48(W1):W449-W454. doi: 10.1093/nar/gkaa379.

Allele	#	Start	End	Length	Core Sequence	Peptide Sequence	Score	Percentile Rank
HLA-DRB1*15:01	1	71	85	15	VIAWNSNNL	TGCVIAWNSNNLDSK	0.9477	0.04
HLA-DRB3*01:01	1	102	116	15	FERDISTEI	LKPFERDISTEIYQA	0.9213	0.08
HLA-DRB3*01:01	1	101	115	15	FERDISTEI	NLKPFERDISTEIYQ	0.9054	0.10
HLA-DRB1*15:01	1	70	84	15	VIAWNSNNL	FTGCVIAWNSNNLDS	0.9068	0.12
HLA-DRB3*01:01	1	103	117	15	FERDISTEI	KPFERDISTEIYQAG	0.8415	0.14
HLA-DRB3*01:01	1	100	114	15	FERDISTEI	SNLKPFERDISTEIY	0.8108	0.16
HLA-DRB1*07:01	1	135	149	15	FQPTYGVGY	SYGFQPTYGVGYQPY	0.7946	0.48
HLA-DRB1*07:01	1	134	148	15	FQPTYGVGY	QSYGFQPTYGVGYQP	0.7993	0.48
HLA-DRB1*15:01	1	72	86	15	VIAWNSNNL	GCVIAWNSNNLDSKV	0.8281	0.48
HLA-DRB3*01:01	1	40	54	15	IRGDEVRQI	SFVIRGDEVRQIAPG	0.6526	0.54
HLA-DRB1*15:01	1	69	83	15	VIAWNSNNL	DFTGCVIAWNSNNLD	0.7811	0.60
HLA-DRB1*03:01	1	40	54	15	IRGDEVRQI	SFVIRGDEVRQIAPG	0.8278	0.67
HLA-DRB3*01:01	1	39	53	15	IRGDEVRQI	DSFVIRGDEVRQIAP	0.5893	0.69
HLA-DRB3*02:02	1	5	19	15	VLYNSASFS	DYSVLYNSASFSTFK	0.5385	0.80
HLA-DRB3*01:01	1	99	113	15	FERDISTEI	KSNLKPFERDISTEI	0.5313	0.81
HLA-DRB1*03:01	1	39	53	15	IRGDEVRQI	DSFVIRGDEVRQIAP	0.7754	0.95

Figure S3. MHC-II Binding Prediction Results. Binding Scores.
Prediction method: NetMHCiipan_el 4.1 | High score = good binders

Peptide Information:
Peptide
ALHMDYSVLYNSASFSTFKCYGVSPTKLNDLCFTNVYADSFVIRGDEVRQIAPGQTGNIADYNYKLPDDFTGCVIAWNSNNLDSKVGGNYNYLYRLFRKSNLKPFERDISTEIYQAGSTPCNGVKGFNCYFPLQSYGFQPTYGVGYQPYRVVVLSFELLHAPATVCGPKKSTNLEFY

MHC-I Alleles:
7-allele HLA reference set
HLA-DRB1*03:01
HLA-DRB1*07:01
HLA-DRB1*15:01
HLA-DRB3*01:01
HLA-DRB3*02:02
HLA-DRB4*01:01
HLA-DRB5*01:01
.Among Europeans, the most common HLA MHC class II cell surface receptor encoded by the human leukocyte antigen (HLA) is HLA-DRB1. HLA-DRB1 is one of the genes in the HLA complex that encodes MHC class II molecules, which play a crucial role in the immune system by presenting antigens to T cells and initiating immune responses. The specific HLA-DRB1 alleles can vary among individuals, and certain alleles may be more common in specific European populations, but HLA-DRB1 is a prominent and diverse group of alleles within the HLA system in Europeans.

Conclusion: Based on the score values, the investigated peptide contains epitopes with a strong predicted binding affinity to HLA-DRB.

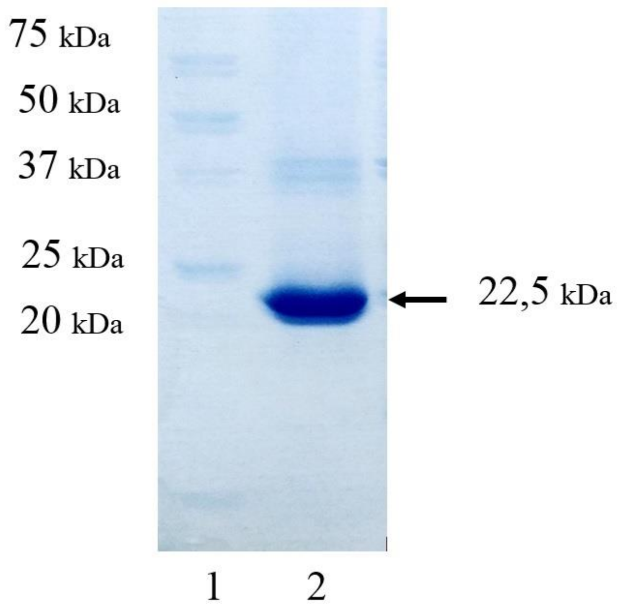

Figure S4. Electrophoresis of SB protein on a 10% polyacrylamide gel. Lane 1: Molecular weight marker (Precision Plus Protein™ Standards Unstained, BioRad, USA); Lane 2: SB protein.
